# Dielectric and Thermal Conductivity Characteristics of Epoxy Resin-Impregnated H-BN/CNF-Modified Insulating Paper

**DOI:** 10.3390/polym12092080

**Published:** 2020-09-13

**Authors:** Hongda Yang, Qingguo Chen, Xinyu Wang, Minghe Chi, Jinfeng Zhang

**Affiliations:** 1 Key Laboratory of Engineering Dielectrics and Its Application, Ministry of Education, Harbin University of Science and Technology, Harbin 150080, China; yanghongda_phd16@hrbust.edu.cn (H.Y.); chiminghe1985@hrbust.edu.cn (M.C.); 2 School of Electrical Engineering and Automation, Qilu University of Technology (Shandong Academy of Sciences), Jinan 250353, China; zhangjinfeng_phd16@hrbust.edu.cn

**Keywords:** dry bushing, epoxy resin-impregnated paper, dielectric characteristics, thermal conductivity, space charge, nanocomposite

## Abstract

High-voltage direct-current (HVDC) dry bushing capacitor-core insulation is composed of epoxy resin-impregnated insulating paper (RIP). To improve the thermal conductivity, breakdown strength, and space charge characteristics of RIP, 0.1 wt % nano-cellulose fiber (CNF)-modified RIP (CNF/RIP), 2.5–30 wt % hexagonal boron nitride (h-BN)-modified RIP (h-BN/RIP), and 2.5–30 wt % h-BN + 0.1 wt % CNF-modified RIP (h-BN + 0.1 wt % CNF/RIP) were prepared. Scanning electron microscopy (SEM) was implemented; the thermal conductivity, DC conductivity, DC breakdown strength, and space charge characteristics were tested. The maximum thermal conductivity of h-BN + 0.1 wt % CNF/RIP was 0.376 W/m.K with a h-BN content of 30 wt %. The thermal conductivity was 85.2% higher than that of unmodified RIP. The breakdown strength and charge suppression were the best in the case of 10 wt % h-BN + 0.1 wt % CNF/RIP. The maximum breakdown strength was 11.2% higher than that of unmodified RIP. These results can play a significant role in the research and development of insulation materials for HVDC dry bushing.

## 1. Introduction

To meet the demand for electricity, attempts are being made to realize high-voltage power transmission with large capacity. Direct-current (DC) transmission is better than alternating current (AC) transmission for asynchronous grid connection; furthermore, power loss is less in the former case. Specifically, for long-distance and under-sea transport, DC transmission is more advantageous than alternating AC transmission [1]. High-voltage direct-current (HVDC) bushing plays an important role in HVDC transmission. To meet the requirements of an oil-free valve hall, environmental friendliness, convenient transportation, a flexible installation angle, and avoidance of secondary injury caused by equipment failure, HVDC dry bushing is gradually replacing the traditional oil-impregnated paper insulation bushing in the HVDC transmission system [2]. The development of HVDC dry bushing is rapid. The capacitor core is the key component of HVDC dry bushing; it features a multi-electrode concentric capacitor structure composed of epoxy resin-impregnated paper (RIP) and aluminum foil. The RIP (for insulation) and aluminum foil play the role of homogenizing the electric field distribution [3].

A large amount of current flowing through the center conductor will result in the capacitor core generating considerable heat. The heat cannot be released in time due to the poor thermal conductivity of RIP. A higher temperature field will form in the insulation of the capacitor core. The DC conductivity of RIP is a function of temperature. In the case of DC voltage, the electric field distribution is closely related to DC conductivity. The temperature gradient will distort the distribution of the electric field in insulation. Due to electric field distortion, the aging of RIP will accelerate, which will reduce the service life of the HVDC dry bushing [1]. Jyothi proposed a method for calculating the temperature distribution in the bushing, which can evaluate the maximum thermal voltage in the bushing insulation [4]. Wang simulated the temperature distribution in the bushing according to the actual operation of the bushing [5]. The influence of bushing structure parameters on temperature distribution is discussed, and the bushing structure is optimized. They provided guidance on the design and manufacture of bushing. Furthermore, the space charge injection can also distort the electric field distribution. In serious cases, the existence of space charges may lead to the failure of bushing insulation. Wu prepared RIP samples and tested their space charge distribution under different electric field strengths [2]; through finite element simulation, Zhang proved that the existence of space charge can distort the electric field distribution in the bushing [6]. Additionally, the breakdown strength of RIP is also an important parameter of dry bushing. Shen, Wei, and Zhang prepared RIP samples and tested their AC and DC breakdown characteristics, which laid a certain foundation for the design and development of high voltage DC bushing [7,8]. The weight and length of a ±1100 kV wall bushing are 18 t and 25 m, respectively. Enhancing the breakdown strength of RIP can significantly reduce the volume and cost of bushing [9]. Therefore, improving the thermal conductivity, space charge characteristic, and breakdown strength of RIP plays a significant role in HVDC dry bushing. The research and development of insulating materials with excellent properties is one of the most direct ways of solving the aforementioned problems.

However, most of the above studies are limited to the performance analysis of the RIP material itself and the optimization of the bushing structure. There are relatively few studies on the modification of RIP insulation materials. Nano-modification is a practicable method of improving the properties of composite materials. Nano-modification of epoxy resin has achieved fruitful results. Tian found that nano-SiO_2_/EP and Al_2_O_3_/EP composites can effectively inhibit the accumulation of space charge in epoxy resin [10]. Song, Park, and Kim showed that graphene/epoxy composites have high thermal conductivity [11]. However, nano-fillers increase the viscosity of epoxy resin, thereby rendering its impregnation difficult. In addition, some scholars have conducted considerable research on oil-impregnated nano-modified insulating paper. Lv found that nano-TiO_2_ can effectively inhibit the space charge injection of oil-impregnated insulating paper [12]. A Bai study found that AlN can effectively inhibit space charge accumulation in oil-impregnated meta-aramid paper [13]. These studies provide a new way to improve the performance of RIP. According to the results of our previously conducted research, hexagonal boron nitride (h-BN) can improve the space charge and thermal conductivity characteristics of RIP [9]. However, the reduced breakdown strength of h-BN/RIP is undesirable. Zeng and Wu used ball milling and scraper orientation methods to obtain h-BN/CNF high thermal conductivity material [14,15]. The nano-cellulose fiber (CNF) features high surface energy, and its structure is similar to that of paper cellulose. Therefore, CNFs are considered a “bridge” (they can be closely combined with pulp cellulose and they can also be entangled with h-BN) between h-BN and paper cellulose to improve their interface. To improve the breakdown strength of h-BN/RIP, h-BN is modified by CNFs. Then, the modified h-BN is used to modify RIP. Moreover, an epoxy anhydride-curing system with excellent insulation performance and low viscosity is used to impregnate insulating paper.

This study investigates the DC dielectric and thermal conductivity characteristics of RIP. Experimental research indicates that 10 wt % h-BN + 0.1 wt % CNFs/RIP feature excellent breakdown strength and space charge suppression.

## 2. Materials and Methods

### 2.1. Sample Preparation

Unbleached coniferous Kraft pulp was used as raw material for the insulating paper. Unmodified insulating paper was produced through the processes of pulping, dissociating, shaping, compressing, and drying.

**Figure 1:**
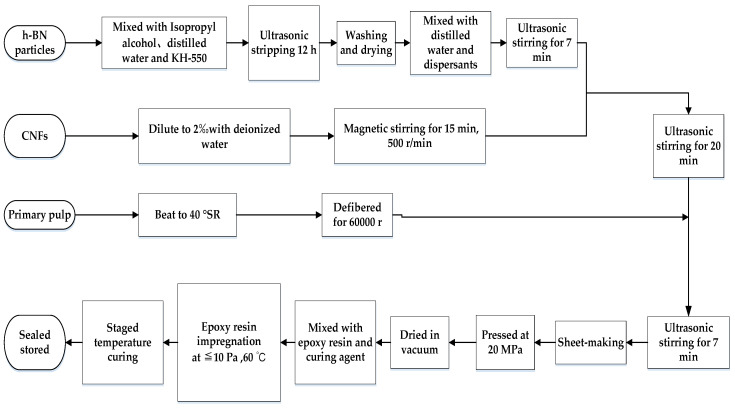
Flow chart of the process of creating epoxy resin-impregnated nano-hexagonal boron nitride (h-BN) + nano-cellulose fiber (CNF)-modified pressboard.

Nano-modified insulating paper was prepared by the Mechanical Co. ultrasonic mixing method. Epoxy resin, curing agent, and accelerator were included in the weight ratio of 100:85:0.3. Then, they were stirred under vacuum at 333.15 K for 15 min. This mixture was used to impregnate the unmodified and modified insulating paper under vacuum at 333.15 K. Lastly, the modified and unmodified RIP were obtained by staged curing of the impregnated insulating paper. The curing conditions are 353.15 K for 2 h, 373.15 K for 2 h, and 393.15 K for 4 h. The process of producing nano-h-BN + CNFs modified RIP is detailed in Figure 1.

Silane coupling agent KH-550 was used as a surface modifier of nano-h-BN, and polyethylene glycol (PEG) with a long chain structure was used as a dispersant [16]. The material parameters and equipment models used in Figure 1 are listed in Table 1.

### 2.2. Measurement System

According to ASMT-E1530, a thermal conductivity test was performed using the thermal conductivity tester (DTC-300, TA Instruments, Newcastle, PA, USA) at 298.15 K [17]. The diameter of the test sample was 50 mm. The thickness of the sample was measured and a thermal paste was applied on both sides of the sample before testing.

A three-terminal electrode system was employed during the DC conductivity measurement. The sample surfaces were aluminized as electrodes. The electric field was 10 kV/mm, and the temperature was maintained at 298.15 K. The electrometer (6517A, Keithley Instruments, Inc., Cleveland, OH, USA) was used to record the stable current (I). The average conductivities samples and the standard deviation of 4 samples were used to evaluate the conductivity characteristics.

A DC breakdown strength test was conducted using a cylindrical electrode, in accordance with ASTM-D149. The temperature was maintained at 298.15 K. The entire test system was placed in transformer oil to avoid surface breakdown [18]. The average breakdown strength and standard deviation of 15 samples were used to evaluate the breakdown performance.

**Figure 2:**
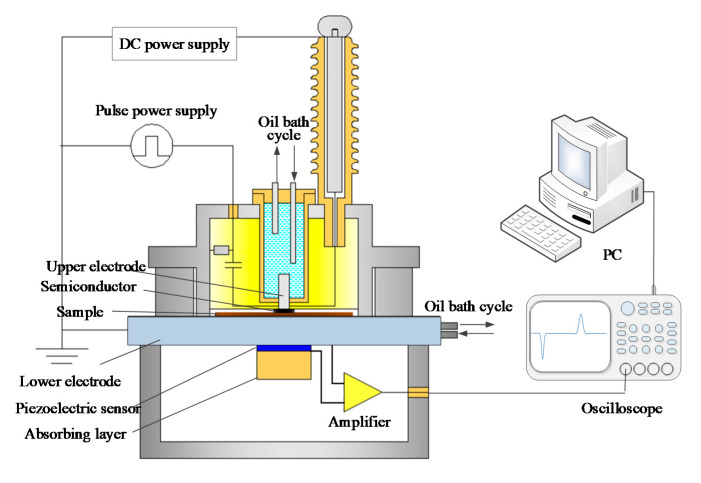
Structure of the pulsed electro-acoustic (PEA) test system.

The pulsed electro-acoustic (PEA) technique was applied during the space charge test. The test field strength was 10 and 20 kV/mm, respectively, and a pulse field strength of 2 kV/mm was applied simultaneously. The test temperature was maintained at 298.15 K. The structure of the PEA test system is illustrated in Figure 2.

**Figure 3:**
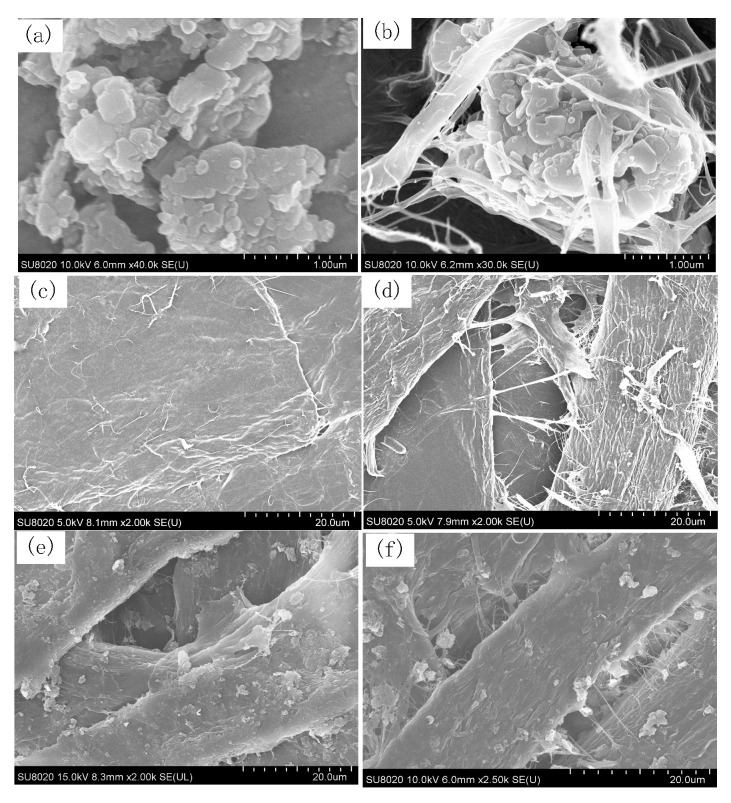
SEM image: (**a**) nano-h-BN sheets, (**b**) CNF-modified nano-h-BN, (**c**) unmodified pressboard, (**d**) CNF-modified pressboard, (**e**) h-BN-modified pressboard, and (**f**) h-BN + CNF-modified pressboard.

The scanning electron microscope (SEM, SU8020 Hitachi High Technologies Corp, Tokyo, Japan) observation results are presented in Figure 3.

## 3. Results

### 3.1. SEM Test Results of the RIP

Figure 3a shows that nano-h-BN is stacked in layers. Figure 3b illustrates nano-h-BN sheets covered with CNFs. CNFs can prevent the agglomeration of h-BN to some extent. Figure 3d shows that there are many nanofibers between pulp cellulose and presents a good combination of CNFs and pulp cellulose. Figure 3e,f shows that CNF-modified h-BN dispersed more evenly in pulp cellulose. It is due to CNFs being able to serve as a “bridge” for strengthening the connection between pulp cellulose and nano-h-BN sheets. Moreover, CNF can separate h-BN from each other.

### 3.2. Thermal Conductivity Test Results of the RIP

**Figure 4:**
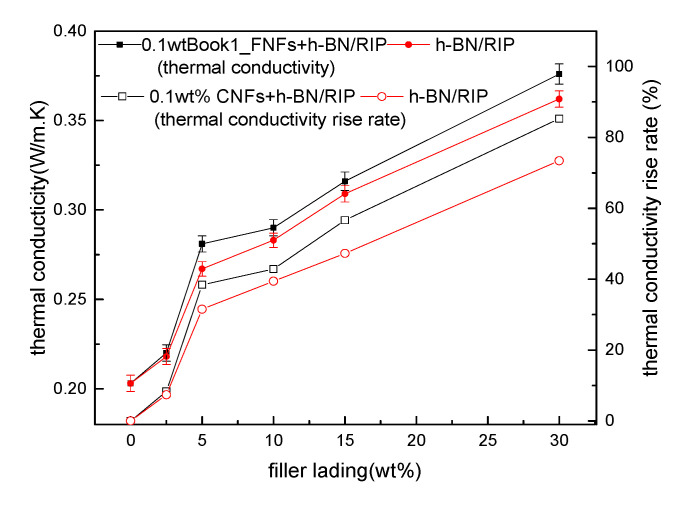
Relationship between thermal conductivity of resin-impregnated insulating paper (RIP) and h-BN content.

The thermal conductivity and rate of rise in thermal conductivity of 0–30 wt % h-BN + 0.1 wt % CNF/RIP and 0–30 wt % h-BN/RIP are presented in Figure 4. When the h-BN content is the same, the thermal conductivity of 0.1 wt % CNFs/RIP is slightly higher than that of h-BN/RIP. The thermal conductivity of 0–30 wt % h-BN + 0.1 wt % CNF/RIP and 0–30 wt % h-BN/RIP increased from 0.203 W/m.K to 0.376 W/m.K and 0.362 W/m.K, respectively. The higher the content of h-BN, the higher the thermal conductivity. The rate of rise in thermal conductivity of 30 wt % h-BN + 0.1 wt % CNFs/RIP and 30 wt % h-BN/RIP reached 85.2% and 73.4% respectively.

The rate of rise in thermal conductivity, *η*, is given by

(1) $$\eta =\;(\lambda_{m}-\lambda_{0})/\lambda_{0}\times 100\%$$

where λ_m_ and λ_0_ are the thermal conductivities of m wt % h-BN + 0.1 wt % CNF/RIP (or m wt % h-BN/RIP) and unmodified RIP, respectively.

### 3.3. DC Conductivity Test Results of The RIP

**Figure 5:**
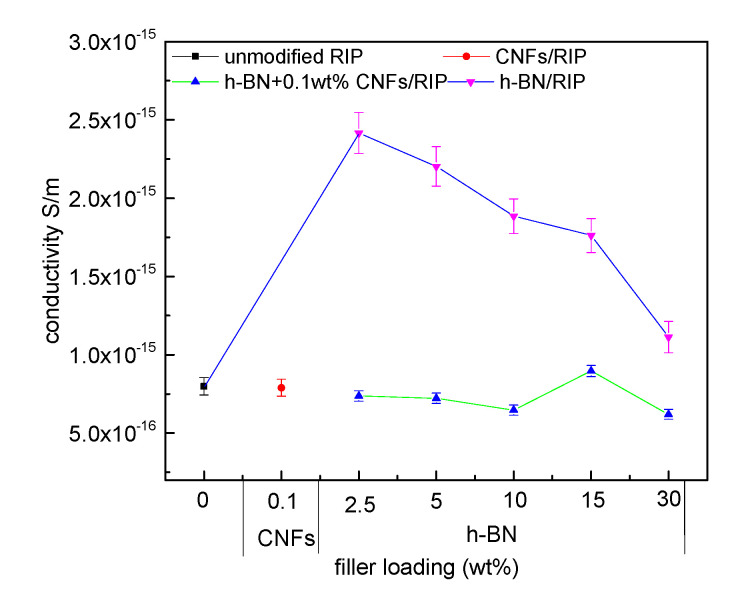
DC conductivity of unmodified RIP, CNF/RIP, h-BN/RIP, and h-BN/0.1 wt % CNF/RIP at 10 kV/mm.

Figure 5 explains the relationship between DC conductivity of unmodified RIP, 0.1 wt % CNF/RIP, 2.5–30 wt % h-BN/RIP, and 2.5–30 wt % h-BN + 0.1 wt % CNF/RIP and h-BN (or CNF) content. The conductivity of CNF/RIP is almost the same as that of unmodified RIP; the DC conductivity of h-BN + 0.1 wt % CNF/RIP is lower than that of h-BN/RIP when the filler loadings of h-BN are the same. As the filler loading of h-BN increases, the DC conductivity of 2.5–30 wt % h-BN/RIP decreases; the DC conductivity of h-BN + 0.1 wt % CNF/RIP is lower than that of unmodified RIP, except for 15 wt % h-BN + 0.1 wt % CNFs/RIP. As the filler loading of h-BN increases, the DC conductivity of 2.5–15 wt % h-BN + 0.1 wt % CNF/RIP first decreases and then increases.

### 3.4. DC Breakdown Strength Test Results of The RIP

**Figure 6:**
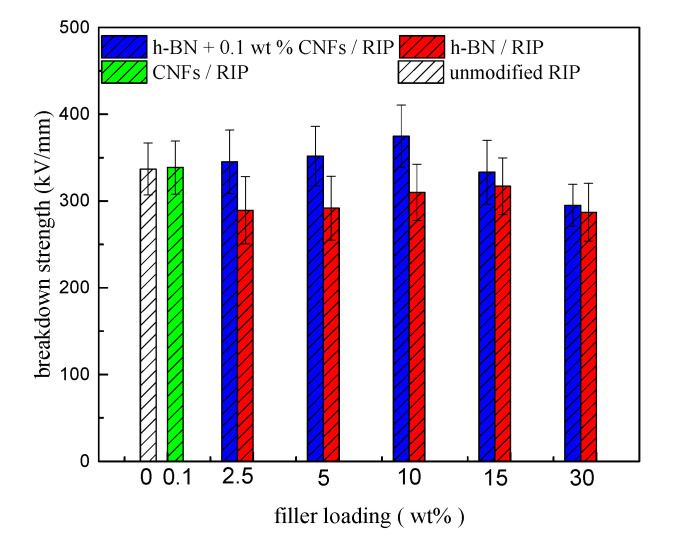
DC breakdown strength of unmodified RIP, BN/RIP, CNF/RIP, and BN + 0.1 wt % CNF/RIP.

Figure 6 presents the results of the DC breakdown strength tests conducted on unmodified RIP, CNF/RIP, h-BN/RIP, and BN + 0.1 wt % CNF/RIP. The breakdown strength of CNF/RIP is almost the same as that of unmodified RIP. When the filler loading of h-BN was in the range of 2.5–30 wt %, the breakdown strengths of h-BN/RIP and h-BN + 0.1 wt % CNF/RIP first increased and then decreased. However, the breakdown strength of h-BN + 0.1 wt % CNF/RIP was higher than that of h-BN/RIP when the contents of h-BN were the same. The breakdown strength of 10 wt % BN + 0.1 wt % CNF/RIP was the highest—11.2% higher than that of unmodified RIP.

### 3.5. Space Charge Characteristics of the RIP

**Figure 7:**
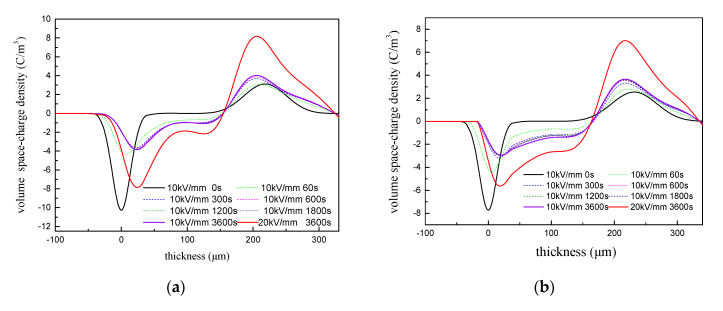

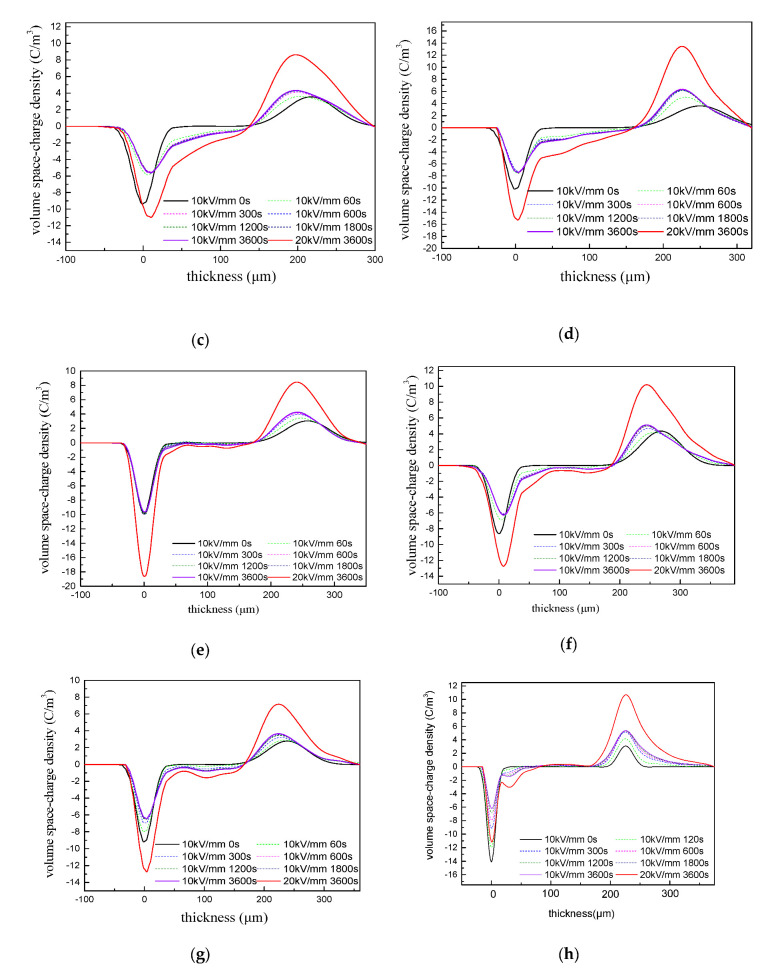
Space charge characteristics of unmodified RIP, CNF/RIP, and h-BN + 0.1 wt % CNF/RIP (**a**) unmodified RIP, (**b**) 0.1 wt % CNF/RIP, (**c**) 2.5 wt % h-BN + 0.1 wt % CNF/RIP, (**d**) 5 wt % h-BN + 0.1 wt % CNF/RIP, (**e**) 10 wt % h-BN + 0.1 wt % CNF/RIP, (**f**) 15 wt % h-BN + 0.1 wt % CNF/RIP, (**g**) 30 wt % h-BN + 0.1 wt % CNF/RIP and (**h**) 10 wt % h-BN/RIP.

The results of the space charge tests conducted on unmodified RIP, CNF/RIP, 10 wt % h-BN/RIP, and h-BN + 0.1 wt % CNF/RIP are presented in Figure 7. Figure 7 shows that with the increase of applied voltage time, the amount of space charge injection increases gradually, the depth deepens, and finally and gradually stabilizes. The figure indicates that more space charges are injected and the injection depth is deeper when the electric field strength is higher. Figure 7a,b indicate considerable space charge injection in the unmodified RIP and CNF/RIP. It can be concluded that only the addition of CNFs cannot inhibit space charge injection. Figure 7a,c–g indicate that space charge injection in h-BN + 0.1 wt % CNF-modified RIP is less than that in the unmodified RIP. However, the amount of charge at the cathode has increased. Furthermore, the space charge injection in 10 wt % h-BN + 0.1 wt % CNF-modified RIP is very little. According to the results in our previous research, only adding h-BN can also suppress space charge injection [1]. Figure 7h shows that 10 wt % h-BN/RIP can suppress space charge injection to some extent. This proves that h-BN plays an important role in suppressing the space charge. However, it was observed that changes in specific mass fraction of h-BN + 0.1 wt % CNF had a more significant effect on space charge suppression than the addition of h-BN alone.

## 4. Discussion

In this section, we shall discuss the thermal conductivities of unmodified RIP and CNF/RIP. The structures of CNFs and insulating paper cellulose are similar. Besides, the CNF content in CNF/RIP is very low. Therefore, adding CNFs alone did not lead to changes in the thermal conductivity of RIP; the thermal conductivity of h-BN + 0.1 wt % CNF/RIP was higher than that of the unmodified RIP. The thermal conductivity of h-BN is very high, and the h-BN in RIP can build a thermal network. Moreover, the higher the h-BN content, the more complete the established thermal network [1]. Therefore, thermal conductivity increases with an increase in the h-BN content. The thermal conductivity of 0.1 wt % CNFs+h-BN/RIP is higher than that of h-BN/RIP, when the h-BN content is the same. It may be due to CNFs improving the interface of h-BN and pulp cellulose, which reduced the interface thermal resistance.

The DC conductivities of unmodified RIP and CNF/RIP are almost the same because their structures are similar and CNF contents are low; the DC conductivity of h-BN/RIP is higher than that of unmodified RIP and decreases with an increase in the filler loading of h-BN. The significant difference between the sizes of h-BN and paper cellulose is a reason why the interface interaction is weak. h-BN affects the conductivity of h-BN/RIP by “scattering” [19]. The scattering effect is weak because the h-BN content is low; furthermore, the adsorption of h-BN on the pulp cellulose will reduce cross-linking points to affect cellulose cross-linking, which can make the movement of the cellulose molecular chain easier. The carrier is easy to transport, so its conductivity increases. As the h-BN content increases, the scattering increases, and the h-BN fills some voids between the pulp cellulose. The carrier transport is inhibited, the conductivity of h-BN/RIP decreases. Therefore, the conductivity of h-BN/RIP increases and then decreases as the filler loading of h-BN increases; the specific surface area and surface energy of CNFs are large, so that they can interact with h-BN. The structures of CNFs and paper cellulose are similar, which makes the CNFs easily combine with insulating paper cellulose. The CNFs can be used as a “bridge” between h-BN and insulating paper cellulose. They can help in combining h-BN and paper cellulose more effectively. In addition, coating h-BN with CNFs can play a supporting role and prevent the agglomeration of h-BN. More interfaces are introduced, and carrier migration is limited [20]. In addition, Figure 3e shows that there are many CNFs linking between the pulp cellulose and pulp cellulose. These CNFs will make the movement of the cellulose molecular chain difficult and fill the voids between the pulp cellulose. At the same time, the presence of CNFs enables h-BN not only to be adsorbed on the pulp cellulose, but also to be fixed between the pulp cellulose by CNFs. The decrease in free volume reduces the free travel of carriers and makes carrier transport difficult. Therefore, the conductivity of h-BN + 0.1 wt % CNF/RIP is lower than those of unmodified RIP and h-BN/RIP. When the filler loading of h-BN is up to 15 wt %, its conductivity increases owing to the weakening of the interface. When the filler loading of h-BN reaches 30 wt %, its conductivity decreases owing to the enhanced “scattering” of h-BN.

For the DC breakdown strength variations in unmodified, CNF/RIP, h-BN/RIP, and h-BN + 0.1 wt % CNF/RIP, the free path of the carriers affects the progress of breakdown. The longer the free path of the carrier, the higher the energy obtained in the process of carrier transmission, and the lower the breakdown strength [9]. The carrier–transport process can be reflected by its DC conductivity. The DC conductivity of h-BN/RIP is higher than those of unmodified RIP and h-BN + 0.1 wt % CNF/RIP, and the carrier transport is easier. As a result, the breakdown strength of h-BN/RIP is lower than those of unmodified RIP and h-BN + 0.1 wt % CNF/RIP. However, although the conductivity of 30 wt % h-BN/RIP is lower than that of 15 wt % h-BN/RIP, its breakdown field strength is lower than that of 15 wt % h-BN/RIP. Because the content of h-BN is very high, some defects are introduced [21]; because the conductivities of unmodified RIP and CNF/RIP are almost the same, the breakdown strengths of RIP and CNF/RIP are almost the same; due to the strong interface effect, the breakdown strength of 2.5–10 wt % h-BN + 0.1 wt % CNF/RIP increases as the h-BN content increases. When h-BN content ≥ 15 wt %, the interface effect becomes weaker. Therefore, the breakdown strength of 15–30 wt % h-BN + 0.1 wt % CNF/RIP decreases.

The space charge characteristics of unmodified RIP, CNF-modified RIP, and h-BN + 0.1 wt % CNF/RIP are affected by the interface and h-BN “scattering”. According to our previous study, appropriate use of h-BN can achieve space charge suppression by “scattering” [19]. In addition, the interface effect also helps in suppressing the space charge. According to the results of DC conductivity and DC breakdown strength, the interface effect is the largest in the case of 10 wt % h-BN + 0.1 wt % CNF/RIP. Therefore, the effect of space charge suppression is the best in the case of 10 wt % h-BN 10 wt % h-BN + 0.1 wt % CNF/RIP. Furthermore, as h-BN continues to increase, the “scattering” becomes stronger. Therefore, 15–30 wt % h-BN + 0.1 wt % CNF/RIP can still achieve space charge suppression.

The 0.1 wt % CNFs+10wt % h-BN/RIP with the thermal conductivity of 0.29 W/m.K, the high breakdown strength of 374.75 kV/mm, the low conductivity of 6.47318^^−16^ S/m, and the good space charge suppression effect is a good choice for HVDC dry bushing insulation.

## 5. Conclusions

This study investigated the dielectric properties of h-BN/RIP, CNF/RIP, and h-BN + 0.1 wt % CNF/RIP. The following conclusions were drawn:

(1) CNF/RIP and h-BN/RIP did not increase the breakdown strength of RIP; however, they did increase the breakdown strength of 2.5–10 wt % h-BN + 0.1 wt % CNF/RIP. h-BN and CNFs were synergetic.
(2) The breakdown and space charge suppression performances were the best in the case of 10 wt % h-BN + 0.1 wt % CNF/RIP. It can be a potential insulating material in the manufacturing of HVDC dry bushing.
(3) In comparison with unmodified RIP, the h-BN/RIP and h-BN + 0.1 wt % CNF/RIP can suppress the space charge but CNF/RIP cannot. h-BN plays a significant role in suppressing the space charge, and CNFs can enhance its role.

## Figures and Tables

**Table 1:** Material parameters and equipment models.

Material or Equipment	Model or Parameter	Manufacturer
Distilled water	µ < 10 S/cm	Prepared in our laboratory
h-BN	Average diameter: 0.5 µm; thickness < 100 nm; purity > 99%	Peng Da Technology Co., Ltd. (Yingkou, China)
Nanocellulose fibers (CNFs)	Diameter: 3–50 nm; length: up to micron; purity > 99%	North Century Technology Research and Development Co., Ltd. (Beijing, China)
Epoxy resin	WSR618 (E-51)	Xingchen Synthetic Material Co., Ltd. (Nantong, China)
Curing agent	Methyl hexahydrophthalic anhydride (MHHPA)	Huicheng Electronic Materials Co., Ltd. (Puyang, China)
Accelerant	2,4,6-Tri(dimethylaminomethyl)phenol (DMP-30)	Shanfeng Chemical Co., Ltd., (Changzhou, China)
Isopropyl alcohol	Analytical purity	Tianjin Fuyu Fine Chemical Co., Ltd. (Tianjin, China)
Coupling agent	KH-550	Nanjing Union Silicon Chemical Co., Ltd. (Nanjing, China)
Beater	TD 6-23	Tongda Light Power Equipment Co., Ltd., Xianyang, China
Ultrasonic cleaning machine	JP-020	Jiemeng Cleaning Equipment Co., Ltd., (Shenzhen, China)
Standard agitator	DJ1C-100	Dadi Automation Instrument, (Jintan, China)
Hand-sheet former	TD10-200	Tongda Light Power Equipment Co., Ltd.,(Xianyang, China)
Curing press	XLB25-D	Shuangli Automation Technology Equipment Co., Ltd., (Huzhou, China)
Vacuum drying chamber	DZF-6210D	Haixiang Instrument and Equipment Factory, (Shanghai China)
Polyethylene glycol (PEG)	Degree of polymerization:2000	Tianjin Guangfu Chemical Research Institute, (Tianjin, China)

## References

[1] Hongda Y., Chen Q., Wang X., Chi M.-H., Liu H., Ning X. (2019). Dielectric and Thermal Conductivity of Epoxy Resin Impregnated Nano-h-BN Modified Insulating Paper. Polymers.

[2] Wen C., Shen W., Wu K. (2015). Study on the properties of space charge and breakdown for epoxy–paper composites. IEEJ Trans. Elect. Electr. Eng..

[3] Ning X., Peng Z., Feng H., Liu P. (2015). Dielectric properties of epoxy and epoxy/creep paper composites dry casings. Chin. J. Electr. Eng..

[4] Jyothi N., Ramu T., Mandlik M. (2010). Temperature distribution in resin impregnated paper insulation for transformer bushings. IEEE Trans. Dielectr. Electr. Insul..

[5] Wang Q., Liao J., Tian H., Peng Z., Liu P. (2017). Regularity analysis of the temperature distribution of epoxy impregnated paper converter transformer bushings. IEEE Trans. Dielectr. Electr. Insul..

[6] Zhang L., Zhang L., Li Q., Zou L., YU C. (2014). Electric field computation of ± 1000 kv dc wall bushing with consideration of space charge effects. Int. Trans. Electr. Energy Syst..

[7] Wei S., Wu K., Cao W. Study on the breakdown and space charge properties of epoxy impregnated paper composites. Proceedings of the 2011 International Symposium on Electrical Insulating Materials.

[8] Zhang S. Experimental study on the electrical and thermal properties of epoxy-crepe paper composites for use in UHV DC bushing condensers. Proceedings of the IEEE International Conference on Solid Dielectrics.

[9] Chen Q., Hongda Y., Wang X., Liu H., Zhou K., Ning X. (2019). Dielectric Properties of Epoxy Resin Impregnated Nano-SiO_2_ Modified Insulating Paper. Polymers.

[10] Tian F.Q., Lin L., Zhang J.L. (2017). Space charge and dielectric behavior of epoxy composite with SiO_2_-Al_2_O_3_ nano-micro fillers at carried temperatures. Compos. Part B Eng..

[11] Song S.H., Park K.H., Kim B.H., Choi Y.W., Jun G.H., Lee N.J., Kong B.-S., Paik K.-W., Jeon S. (2012). Enhanced Thermal Conductivity of Epoxy-Graphene Composites by Using Non-Oxidized Graphene Flakes with Non-Covalent Functionalization. Adv. Mater..

[12] Lv C., Liao R.J., Wu W. (2015). Influence of Nano-TiO_2_ on DC Space Characteristics of Oil-paper Insulation Material. High. Volt. Eng..

[13] Bai G.R., Liao J., Liu N. (2015). Influence of Nano-AlN Modification on the Dielectric Properties of Meta-aramid Paper. High. Volt. Eng..

[14] Wu K., Fang J., Ma J., Huang R., Chai S., Chen F., Fu Q. (2017). Achieving a Collapsible, Strong, and Highly Thermally Conductive Film Based on Oriented Functionalized Boron Nitride Nanosheets and Cellulose Nanofiber. ACS Appl. Mater. Interfaces.

[15] Chen L., Xiao C., Tang Y., Zhang X., Zheng K., Tian X. (2019). Preparation and properties of boron nitride nanosheets/cellulose nanofiber shear-oriented films with high thermal conductivity. Ceram. Int..

[16] Chen Q., Liu H., Chi M., Wang Y., Wei X. (2017). Experimental Study on Influence of Trap Parameters on Dielectric Characteristics of Nano-Modified Insulation Pressboard. Materials.

[17] Meleshenko V.N., Ogon’Kov V.G., Chirikov A.V., Serebryannikov S.V., Cherkasov A.P. (2017). The thermal conductivity of electrical-insulating materials and insulation systems. Russ. Electr. Eng..

[18] Shang N., Chen Q., Wei X. (2018). Preparation and Dielectric Properties of SiC/LSR Nanocomposites for Insulation of High Voltage Direct Current Cable Accessories. Mater. Chem. Phys..

[19] Huang X.Y., Zhang Q., Jiang P.K. (2016). The effect of nanoparticle shape on the electrical properties of linear low density polyethylene nanocomposites. Proc. CSEE.

[20] Wang X., Nelson J., Schadler L., Hillborg H. (2010). Mechanisms leading to nonlinear electrical response of a nano p-SiC/silicone rubber composite. IEEE Trans. Dielectr. Electr. Insul..

[21] Wang W., Min D., Li S. (2016). Understanding the conduction and breakdown properties of polyethylene nanodielectrics: Effect of deep traps. IEEE Trans. Dielectr. Electr. Insul..

